# Emerging and Re-Emerging Parasitic Infections of the Central Nervous System (CNS) in Europe

**DOI:** 10.3390/idr15060062

**Published:** 2023-10-25

**Authors:** Varol Tunali, Metin Korkmaz

**Affiliations:** 1 Department of Parasitology, Faculty of Medicine, Manisa Celal Bayar University, 45030 Manisa, Turkey; 2 Department of Emergency Medicine, Izmir Metropolitan Municipality Eşrefpaşa Hospital, 35170 Izmir, Turkey; 3 Department of Parasitology, Faculty of Medicine, Ege University, 35100 Izmir, Turkey; metin.korkmaz@ege.edu.tr

**Keywords:** protozoan infections, helminthiasis, infectious diseases, travel medicine, human migration, climate change, one health

## Abstract

In a rapidly evolving global landscape characterized by increased international travel, migration, and ecological shifts, this study sheds light on the emergence of protozoal and helminthic infections targeting the central nervous system (CNS) within Europe. Despite being traditionally associated with tropical regions, these infections are progressively becoming more prevalent in non-endemic areas. By scrutinizing the inherent risks, potential outcomes, and attendant challenges, this study underscores the intricate interplay between diagnostic limitations, susceptibility of specific population subsets, and the profound influence of climate fluctuations. The contemporary interconnectedness of societies serves as a conduit for introducing and establishing these infections, warranting comprehensive assessment. This study emphasizes the pivotal role of heightened clinician vigilance, judicious public health interventions, and synergistic research collaborations to mitigate the potential consequences of these infections. Though rare, their profound impact on morbidity and mortality underscores the collective urgency required to safeguard the neurological well-being of the European populace. Through this multifaceted approach, Europe can effectively navigate the complex terrain posed with these emergent infections.

## 1. Introduction

The shifting landscape of parasitic infections affecting the central nervous system (CNS) is challenging the established paradigms in Europe. While traditionally confined to low- and middle-income countries, these infections are now encroaching upon non-endemic regions, propelled with escalating international travel, immunosuppression trends, and climatic variations [1,2]. The augmentation of risk via prolonged immunosuppression and immunosuppressive medications accentuates the gravity of the situation [3]. However, the limited availability of empirical data, due to underreporting, underscores the exigency for a prompt and comprehensive comprehension of this evolving scenario [4].

CNS parasitic infections, such as neurocysticercosis, represent a growing global health concern, particularly in developing nations, where it is the leading cause of preventable epilepsy [5]. Yet, obtaining accurate global estimates of the prevalence and burden of CNS parasitic infections remains challenging due to limited population-wide data, the vast heterogeneity in infection types and locations, and a suspected underdiagnosis and underreporting in resource-limited regions [6].

In the European context, the evolving dynamics of CNS parasitic infections have catalyzed a compelling public health concern. Transformative variables including global tourism, migratory fluxes, and the HIV/AIDS pandemic have expedited the geographical dissemination of these infections, necessitating recalibration of the epidemiological fabric [1,7,8]. Diverse parasites, encompassing protozoa and helminths of the cestode, nematode, and trematode classes, collectively contribute to intricate clinical manifestations spanning subacute meningitis, encephalitis, cerebral lesions, vascular events, and myelopathy [3]. Diagnostic endeavors are obfuscated with the nebulous symptomatology and the unreliability of conventional serological assays, while therapeutic interventions confront the absence of standardized regimens [9].

In this intricate scenario, heightened clinician awareness is crucial [9]. As primary healthcare providers, clinicians play a key role in vigilantly monitoring common and uncommon cases. It is essential for them to be alert to subtle and unusual symptoms while considering factors like international travel and weakened immune systems as potential risks [3]. In this context, this article aims to emphasize the pressing nature of the situation. It provides insights to improve clinical understanding and healthcare practices, effectively addressing the increasing threat posed with documented and potential CNS parasitic infections in Europe.

## 2. Parasitic Infections of the CNS

### 2.1. Protozoal Infections of CNS

#### 2.1.1. Amebiasis (*Entamoeba histolytica*)

Amebiasis, caused by *Entamoeba histolytica*, presents a global health challenge, particularly in regions with inadequate sanitation. Endemic areas in Africa, Asia, and Latin America report an estimated annual burden of around 50 million symptomatic cases, accompanied with a considerable number of asymptomatic carriers [10]. GeoSentinel Surveillance Network data underscore *E. histolytica* as the third most common pathogen among travelers returning with infectious gastrointestinal disease, accounting for 12.5% of confirmed cases and an estimated incidence of 14 cases per 1000 returning travelers [11]. In parts of Asia, Europe, North America, and Australia, specific populations, including gays, bisexuals, and other men who have sex with men (MSM), are identified as being at higher risk of acquiring amebiasis [12]. In Europe, while relatively infrequent, instances of amebiasis are often linked to travel, immigration, or localized transmission, necessitating vigilant surveillance and strategic intervention strategies [12]. There have been reports of brain abscesses caused by *E. histolytica* from Turkey and Spain in Europe [13,14] (Table 1). 

The pathogenesis of an amoebic brain abscess intricately involves *E. histolytica*’s interactions with the host’s immune system and brain tissue [15]. Trophozoites originating from the intestines, following hematogenous spread, reach distant locations, including the brain. By adhering to endothelial cells and overcoming the blood–brain barrier, these trophozoites infiltrate brain tissue [16]. Subsequent immune responses elicit inflammation and tissue damage [17]. The culmination of these events leads to the formation of pus-filled abscesses within the brain. The resultant abscess formation underscores severe clinical manifestations, including severe headaches, fever, seizures, and neurological deficits [15]. A timely diagnosis is pivotal in curbing brain tissue damage and potential complications associated with this life-threatening condition.

Diagnosing amoebic brain abscesses involves a multifaceted approach, including clinical evaluation, imaging techniques, and laboratory confirmation [16]. Identification of *E. histolytica* trophozoites in brain tissue samples, obtained via biopsy or surgical drainage, confirms the diagnosis [12]. Efficient management entails a combined pharmacological approach and surgical intervention if needed. Metronidazole, effective against E. histolytica, is coupled with other antibiotics to address potential bacterial infections [16]. Surgical drainage alleviates pressure and removes infected tissue, warranting neurosurgical consultation and close monitoring.

#### 2.1.2. Free-Living Amebiasis

Free-living amoebae (FLA), including *Naegleria fowleri*, *Acanthamoeba* spp., *Balamuthia mandrillaris*, and *Sappinia*, cause severe CNS diseases and infections in humans and animals [18]. FLA are found in water and soil worldwide, with infections often linked to water exposure or contact lenses. Since its establishment in 1962, the CDC has conducted passive surveillance for Primary Amebic Meningoencephalitis (PAM) in the United States, with 145 reported cases largely originating from southern states, while globally, Naegleria fowleri, the amoeba responsible for PAM, has been detected on every continent except Antarctica, with recent estimates of 235 to 260 reported cases worldwide [19]. In Europe, PAM cases have been primarily associated with locations like natural warm springs in the City of Bath, United Kingdom, and artificially heated areas such as power stations and baths in Belgium and the Czech Republic [20].

FLA’s complex pathogenesis involves trophozoites and cyst stages, cytopathic effects, invasion, and immune responses [21]. The most severe FLA infections involve the CNS. Granulomatous amoebic encephalitis (GAE) and primary amoebic meningoencephalitis (PAM) are the main categories [18]. GAE is characterized by subacute to chronic neurological symptoms, including headache, visual disturbances, neurological deficits, and coma, with a high mortality rate. PAM, caused by *N. fowleri*, progresses rapidly with flu-like symptoms, followed by severe neurological signs and death within days [18]. Differential diagnoses vary based on the site of infection and risk factors. Although rare, cases of GAE, PAM, and keratitis associated with the FLA have been reported in Europe [22,23,24].

The diagnosis of FLA infections relies on microscopy, histopathology, and molecular tests. Treatment effectiveness is highest when administered early in the course of the disease. Combination antimicrobial therapies are often used, with miltefosine showing promise in treatment [25]. In Europe, FLA infections are less common than in some other regions, and there is a focus on awareness, prevention, and a prompt diagnosis. Surveillance, awareness campaigns, and proper contact lens hygiene education are important in managing these rare but serious infections.

#### 2.1.3. Cerebral Malaria

Cerebral malaria is a severe complication of *Plasmodium falciparum* infection and is characterized by the sequestration of infected red blood cells (iRBCs) in the cerebral microvasculature, leading to endothelial activation, inflammation, and disruption of the blood–brain barrier [26]. The pathogenesis of cerebral malaria involves a complex interplay between parasite-related factors, host immune responses, and endothelial dysfunction. One of the key mechanisms in the development of cerebral malaria is the sequestration of iRBCs in the cerebral microvasculature. This sequestration leads to the activation of endothelial cells, triggering the release of pro-inflammatory cytokines, chemokines, and adhesion molecules [27]. This inflammatory response contributes to the recruitment and activation of immune cells, including monocytes, T cells, and platelets. These cells release additional inflammatory mediators, amplifying the immune response and exacerbating endothelial dysfunction [28]. Endothelial dysfunction and disruption of the blood–brain barrier further contribute to the pathogenesis of cerebral malaria. Impaired endothelial function results in increased vascular permeability, allowing the leakage of fluid and proteins into the brain parenchyma, leading to cerebral edema and increased intracranial pressure [29].

According to a Eurosurveillance report, malaria stood out as the predominant arthropod-borne ailment among travelers from Africa, with 34,235 reported cases (with an incidence rate of 28.8 per 100,000 travelers) between 2015 and 2019, exhibiting a steady rise in the number of cases during this period except for a decline in 2016, while the case fatality ratio consistently remained below 1% [30]. According to a meta-analysis, 13.2% of the imported malaria cases in Europe were allocated as severe malaria, with a 6.3% prevalence of deaths among severe malaria patients. Asia, on the other hand, had the highest proportion of deaths from severe imported malaria, followed by Europe [31]. Between 2006 and 2014, a multicenter study conducted by The European Network for Tropical Medicine and Travel Health (TropNet) analyzed 185 patients with severe malaria treated in 12 European countries, reporting that 46 (25%) of these patients presented with cerebral malaria, which was found to be associated with older age [32]. 

A retrospective study conducted at the Hospital for Tropical Diseases in London, United Kingdom, included 124 patients with severe falciparum malaria admitted to the ICU, revealing that cerebral malaria was the most prevalent condition, and both cerebral malaria and acute kidney injury were observed earlier (median day 1) compared to acute respiratory distress syndrome (median day 3) [33]. According to a recent report from Switzerland, malaria cases in Switzerland primarily originate from West Africa, especially among travelers, indicating the need for focused travel medicine efforts in that region, while potential future waves of migrants from Afghanistan and climate change could contribute to the establishment of *P. vivax* malaria transmission in Switzerland, and post-pandemic travel trends may increase malaria cases [34].

The debated post-malaria neurological syndrome (PMNS), which was defined in 1997 by Nguyen et al., is another manifestation of *P. falciparum* in the CNS. This syndrome is characterized by the emergence of encephalitic signs in patients after a symptom-free period (with a median time of 96 h) following a malaria cure, and, notably, these patients have negative blood smears for *P. falciparum* and negative results in investigations for other potential causes [35]. Despite its association with mefloquine treatment by Nguyen et al., several reports from Europe challenge the notion of an association or causative effect between mefloquine and the condition, instead highlighting numerous cases treated with quinine and artemisinin [36,37].

#### 2.1.4. Toxoplasmosis

**Figure 1:**
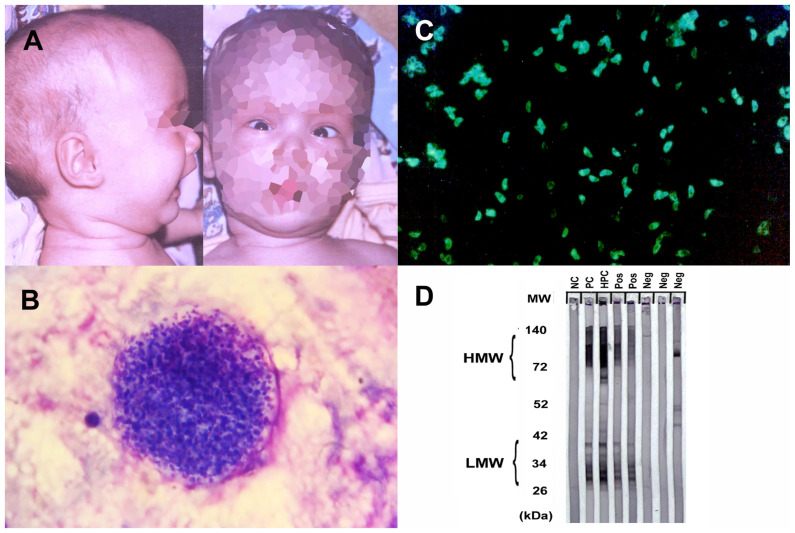
Physical examination and serological findings of some parasitic CNS infections. (**A**) Hydrocephalus and sunset eye sign due to congenital toxoplasmosis. (**B**) *Toxoplasma gondii* latent tissue cysts in the brain, which, enclosing hundreds of bradyzoites, may remain throughout the life and reactivation of these bradyzoites may lead to fatal toxoplasmic encephalitis. (**C**) *Toxoplasma* tachyzoites with bright diffuse or peripheral fluorescence in indirect immunofluorescence assay (IFA) analysis is considered as positive for anti-*Toxoplasma* antibodies. (**D**) Western blot testing for toxocariasis. The first line (NC) to the left is a negative control, the second and third lines (PC, HPC) are positive and high-positive controls, respectively, and the rest are patient samples. Bands of lower molecular weight (LMW) are specific for anti-*Toxocara* antibodies and considered positive for *Toxocara* serology. HMW, high molecular weight; MW, molecular weight; kDa, kilodalton; Pos, positive; Neg, negative).

*Toxoplasma gondii* is a parasite that causes zoonotic infections in humans and infects a wide range of animals. While it usually only results in mild illness in healthy individuals, toxoplasmosis can be a common opportunistic infection with high mortality in immunocompromised individuals, often due to reactivation of infection in the CNS [38]. During the acute phase of infection, interferon-dependent immune responses control rapid parasite expansion and mitigate acute disease symptoms. However, after dissemination, the parasite differentiates into semi-dormant cysts within muscle cells and neurons, where they persist for life in the infected host [38] (Figure 1). *T. gondii* uses various strategies to breach the blood–brain barrier and enter the CNS. These mechanisms include potential Trojan Horse-like entry via infected monocytes and efficient transmigration of extracellular tachyzoites, which may be the primary mode of parasite entry, ultimately leading to the formation of tissue cysts in the CNS. The mechanisms of reactivation are not fully understood but it is thought that it may be due to a decline in cell-mediated immunity. Recent studies suggest that cellular stress is a key factor not only in prompting development of bradyzoites but also in maintaining the encysted form. In immunocompromised patients, reactivation of Toxoplasma infection is typically seen after the CD4+ T-cell count drops below 100–200 cells/mm^3^ [38]. 

Currently, it is reported that 30% of the world’s population has antibodies against *T. gondii* and the estimated pooled prevalence of *T. gondii* infection in people with HIV infection is 35.8% overall, with specific rates of 30.1% in Western and Central Europe [39]. Toxoplasma encephalitis and Toxoplasma retinitis, present in approximately 30% of this risk group, were considered AIDS-defining opportunistic infections before the introduction of highly active antiretroviral therapy (HAART). Even today, toxoplasmosis remains the leading cause of neurological disease in HIV-positive patients, often leading to severe pathology or fatal outcomes [40,41]. Patients with CNS toxoplasmosis commonly experience one or more CNS mass lesions, resulting in symptoms such as headache, confusion, lethargy, changes in consciousness, convulsions, paralysis, emotional dysregulations, poor coordination, muscle weakness, seizures, and alterations in alertness [39].

In addition, several studies have suggested a potential association between toxoplasmosis and mental health disorders, particularly schizophrenia and suicidal behavior. Torrey et al. found a higher prevalence of *T. gondii* infection among individuals with schizophrenia, indicating a possible link between the parasite and schizophrenia symptoms [42]. Additionally, Sutterland et al. conducted a systematic review that provided evidence of an association between toxoplasmosis and suicide attempts [43].

CNS toxoplasmosis affects immunocompromised individuals and requires a swift diagnosis and treatment to prevent severe neurological complications. The diagnosis involves neuroimaging, revealing characteristic ring-enhancing lesions on MRI, alongside serological tests and a CSF analysis for antibodies and DNA detection [44] (Figure 1). 

Treatment combines antimicrobial therapy and immune restoration. Pyrimethamine and sulfadiazine form the core of antimicrobial treatment, supplemented with leucovorin to mitigate side effects. Corticosteroids might be used to control inflammation. Maintaining lower doses of antimicrobial drugs prevents relapse. Immune function restoration, often through antiretroviral therapy for HIV/AIDS patients, addresses the underlying immune deficiency. Regular monitoring is essential, and early intervention coupled with immune management generally leads to a favorable prognosis [45].

#### 2.1.5. Trypanosomiasis

The pathophysiology of trypanosomiasis in the CNS differs depending on the type and stage of the infection. In Human African trypanosomiasis (HAT), caused by *Trypanosoma brucei* species, the parasites undergo a complex life cycle in the tsetse fly vector, where they multiply and develop into epimastigotes in the salivary glands. These forms are injected into the human bloodstream during a tsetse fly bite, where they differentiate into trypomastigotes and rapidly divide with binary fission [46]. The parasites evade the host immune system by changing their variant surface glycoproteins (VSGs), which are exposed on their plasma membrane and elicit a strong antibody response. The parasites disseminate through the bloodstream and lymphatics, reaching various organs and tissues, including the skin, spleen, liver, heart, kidneys, and eyes. Eventually, the parasites cross the blood–brain barrier into the CNS, causing the meningo-encephalitic or second stage [47]. The mechanisms with which the parasites invade the CNS are not fully understood but may involve direct transcytosis across endothelial cells, paracellular migration through tight junctions, or Trojan Horse mechanisms involving infected immune cells [48]. Once in the CNS, the parasites induce a progressive neuroinflammation that involves the activation of microglia and astrocytes, the production of pro-inflammatory cytokines and chemokines, the recruitment of peripheral immune cells, and the disruption of the blood–brain barrier integrity. These changes underlie the altered behavior in the late or secondary disease stages, prevalent in the chronic Gambian form, characterized by hypersomnia leading, and if untreated or if treatment is followed by reactive changes, to coma and death [47]. Reported from more than 20 countries in Africa, in 2015, a total of 2804 cases of HAT were reported to WHO, with the majority (90%) caused by *T. brucei gambiense*, marking a significant 90% reduction since 1999 [49].

In American trypanosomiasis (Chagas disease, CD), caused by *Trypanosoma cruzi*, the infection can be divided into three stages: acute, intermediate, and chronic. The parasites are transmitted by blood-sucking bugs of subfamily Triatominae that defecate on the skin after biting and depositing metacyclic trypomastigotes on it [50]. The parasites enter through mucous membranes or skin abrasions and invade various cell types, including muscle cells, macrophages, and neurons. The parasites differentiate into amastigotes and multiply intracellularly until they lyse the host cell and release trypomastigotes that can infect new cells or enter the bloodstream [50]. In the acute stage, which lasts for 4 to 8 weeks after infection, the parasite produces direct destructive and inflammatory changes in various organs and tissues, including the CNS. The CNS involvement can manifest as meningoencephalitis or chagomas (granulomatous lesions) that can be life-threatening, but which normally resolve spontaneously or with treatment [47]. The intermediate stage is a prolonged asymptomatic period that can last for years or decades, during which most parasites are suppressed by the host immune system and only low levels of parasitemia are detected. However, some parasites may persist in tissues such as cardiac muscle or neurons and cause chronic damage [51]. Characterized by alterations in progressive peripheral neuroimmunopathology, the chronic stage involves autoimmune destruction of various nerve components, particularly the autonomic innervation of the heart and gut. This results in cardiomyopathy or mega syndromes (such as esophagus or colon dilation), affecting approximately 30% of individuals with chronic infections [52]. CD remains endemic in 21 countries of continental Latin America, and globally, its prevalence decreased by 11.3%, declining from an estimated 7,292,889 cases in 1990 to 6,469,283 cases in 2019 [53].

CD and HAT have surfaced in Europe primarily due to migration from endemic regions. Countries like Spain, Portugal, Italy, France, the United Kingdom, and Switzerland, which host substantial migrant populations from CD-endemic areas, have encountered the impact of CD [54]. Spain, in particular, has documented CD cases among diverse patient groups, including those with HIV infection and rheumatologic disorders, transplant recipients, and cancer patients [55]. Europe has also experienced imported HAT cases, with historical data dating back to 1904–1963 and recent reports in countries like France, Italy, Spain, the United Kingdom, Germany, the Netherlands, Belgium, Norway, Sweden, Switzerland, and Poland [56].

**Table 1:** Epidemiology, mode of transmission, diagnosis, and treatment of protozoal diseases of the CNS.

Parasites and Diseases	Countries with Reported Cases (Europe)	Mode of Transmission	Diagnosis	Treatment
*Entamoeba histolytica* Amebiasis	Turkey, Spain [13,14]	Ingestion of cysts	Radiology, serology, molecular	Metronidazole, surgical drainage
Free living amoeba *Naegleria fowleri* *Acanthamoeba* spp. *Balamuthia mandrillaris* *Sappinia*	Belgium, Czech Republic, Italy, the Netherlands, United Kingdom [20,22,23,24]	Trophozoites through nasal passage, olfactory nerve Cysts or trophozoites through eye, nasal passage, lung, or skin	Microscopy, molecular	Symptomatic
*Plasmodium falciparum* Cerebral malaria	United Kingdom, Switzerland [33,34], Italy, Germany, France, Denmark, Belgium [32]	Mosquito bite	Microscopy, molecular	Quinine and artemisinin
*Toxoplasma gondii* Toxoplasmosis	France, Spain, Czech Republic, United Kingdom, Germany, Denmark, Serbia [39]	Ingestion of oocysts or tissue cysts	Radiology, serology	Pyrimethamine and sulfadiazine
*Trypanosoma brucei* African trypanosomiasis (HAT)	France, Italy, Spain, the United Kingdom, Germany, the Netherlands, Belgium, Norway, Sweden, Switzerland, Poland [56]	Tsetse fly bite	Microscopy, molecular	Pentamidine, eflornithine, nifurtimox, melarsoprol, suramin
*Trypanosoma cruzi* South American trypanosomiasis (Chagas Disease (CD))	Spain, Portugal, Italy, France, the United Kingdom, Switzerland [54]	Metacyclic trypomastigotes through mucous membranes or skin abrasions	Microscopy, molecular	Nifurtimox, benznidazole

### 2.2. Helminth Infections of CNS

#### 2.2.1. Angiostrongyliasis

Neural angiostrongyliasis, an emerging zoonotic disease caused by the rat lungworm *Angiostrongylus cantonensis*, poses a growing concern due to its potential to cause eosinophilic meningitis and severe CNS disorders in humans. Angiostrongyliasis has been reported in more than 2800 human cases, with likely underreporting, and its geographical range is expanding, now encompassing Southeast Asia, the Pacific Islands, parts of South and Central America, the Caribbean, Australia, and the United States [57]. Initially considered non-endemic in Europe, the paradigm shifted in 2018 when *A. cantonensis* worms were discovered on the Mediterranean island of Mallorca, Spain, a popular tourist destination [58]. This discovery signaled the possible entry of the rat lungworm into mainland Europe and led to increased surveillance efforts.

The transmission of *A. cantonensis* occurs through a complex life cycle involving definitive hosts, rats, and intermediate hosts, snails, and slugs. Rodents, especially rats, are the primary hosts, acquiring the parasite by consuming third-stage larvae in snails or slugs. These larvae progress through rodents’ respiratory and digestive tracts, eventually being excreted as first-stage larvae. Intermediate hosts like snails and slugs ingest these first-stage larvae, allowing them to mature into third-stage larvae. These third-stage larvae can be transmitted to paratenic hosts, including freshwater shrimps, land crabs, frogs, toads, monitor lizards, and planarians, when these paratenic hosts ingest the infected intermediate hosts. Humans can become infected by consuming paratenic or intermediate hosts with third-stage larvae or via contact with contaminated items, such as vegetables [59]. Over the past several decades, *A. cantonensis* has rapidly expanded its geographical distribution beyond its original Asian range, mainly facilitated by rats that serve as its definitive hosts [60]. These rats often accompany ships and human activities, effectively aiding the dispersion of the parasite to tropical and subtropical regions worldwide [61].

The initial finding of *A. cantonensis* in the sewer system of Valencia, Spain, marked its presence in Continental Europe [62]. Subsequent investigations revealed the parasite’s presence in both *Rattus norvegicus* and *Rattus rattus*, with higher prevalence noted in rats residing in orchards surrounding the city [62]. These orchards contribute to the global distribution of *A. cantonensis* by exporting vegetables to various regions, potentially exposing populations elsewhere to the parasite [62].

Angiostrongyliasis primarily affects the CNS and can lead to neurological symptoms due to the migration of larvae within the brain and spinal cord. The larvae can cause inflammation, tissue damage, and immune responses, resulting in various neurological manifestations [59].

Neurological manifestations of *A. cantonensis* infection include eosinophilic meningitis, encephalitis/encephalomyelitis, radiculitis, cranial nerve abnormalities, and ataxia. Adults with CNS symptoms commonly present severe headaches, neck stiffness, and paraesthesias, while children exhibit nausea, vomiting, somnolence, fever, and muscle twitching [63]. Encephalitis is associated with mental status changes, focal neurological signs, and abdominal pain, often evolving into sensory and motor disturbances, and is more prevalent in children and the elderly. The incubation period varies, and the severity of the disease can depend on the worm burden and the intermediate host [63,64].

Diagnosing angiostrongyliasis often involves a combination of clinical evaluation, examination of cerebrospinal fluid, and imaging studies such as MRI. The presence of eosinophilia and evidence of inflammation in the CNS can support the diagnosis [65]. Treatment may include supportive care to manage symptoms and reduce inflammation [63]. In severe cases, corticosteroids may be used to control the immune response [66]. There is no specific antiparasitic drug for angiostrongyliasis, but the infection is typically self-limiting in many cases.

The epidemiology of Angiostrongylosis in Europe has been demonstrated through reported cases in various countries including France, Germany, the Netherlands, Switzerland, Belgium, Croatia, Italy, Spain, and the United Kingdom [59] (Table 2). In New Caledonia, a retrospective study of eosinophilic meningitis cases revealed that 17 out of 92 cases were confirmed as angiostrongyliasis between 2004 and 2019, predominantly affecting young adults and non-walking infants [65].

#### 2.2.2. Echinococcosis

Echinococcosis, caused by *Echinococcus granulosus* and *Echinococcus multilocularis*, presents diverse epidemiological dynamics in Europe, intertwining human and animal health. These parasitic infections underscore the complex interplay between host populations, transmission patterns, and pathogenesis mechanisms within the continent.

Cystic echinococcosis (CE), primarily caused by *E. granulosus*, exhibits varying prevalence across Europe. Endemic regions encompass Mediterranean countries like Greece, Italy, and Turkey, as well as eastern European nations such as Romania and Bulgaria [67]. Human infections stem from the accidental ingestion of eggs shed by canids, notably dogs. Autochthonous occurrences of *E. multilocularis*, responsible for alveolar echinococcosis (AE), are confirmed in regions including the Baltic states, Belgium, the Netherlands, Italy, Austria, Hungary, and Slovenia [67,68]. High-risk areas often align with environments where intermediate hosts thrive.

*E. granulosus* infects humans through the oral route, with ingested eggs releasing oncospheres that penetrate the intestinal wall and reach various organs, primarily the liver and lungs. In rare cases, oncospheres may reach the CNS via the bloodstream, leading to cyst formation in the spine or surrounding tissues [69]. The host’s immune response to the developing cysts often leads to fibrous encapsulation, attempting to contain the infection [70].

**Figure 2:**
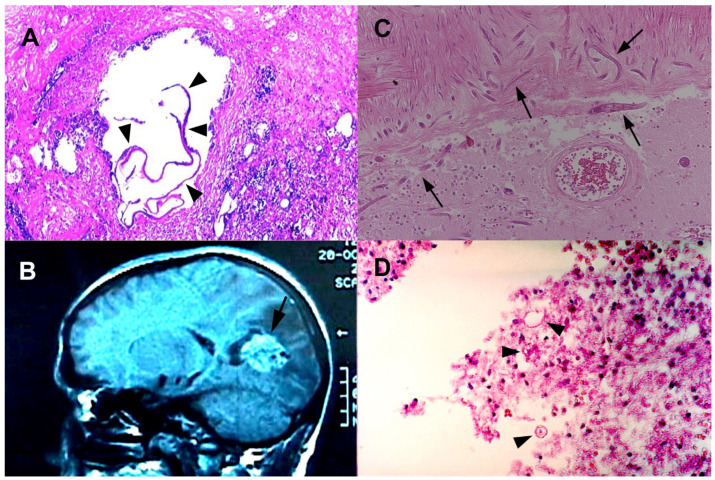
Histopathological and radiological findings of some of the helminth infections of CNS. (**A**) Histopathological examination (Hematoxylin and Eosin stain) of brain lesions shows typical thin and conglomerated laminated membrane (arrowheads) surrounded by necrotic material in an alveolar echinococcosis patient. (**B**) MRI image of circumscribed, tumor-like AE lesion (arrow) in the brain. (**C**) Histopathological image (H&E stain) of brain shows numerous cross-sections of *Strongyloides stercoralis* larvae (arrows). (**D**) *Toxocara* spp. larvae (arrowheads) in the brain of a male patient (H&E stain).

For *E. multilocularis*, the larval stage infiltrates the intermediate host’s organs, forming invasive, multi-vesicular lesions. Upon ingestion of eggs, humans become accidental hosts. *E. multilocularis* can disseminate hematogenously, with the potential to develop metastatic lesions in various organs, including the CNS [71]. In the CNS, these parasites trigger host immune responses, leading to granulomatous inflammation and potential neurological symptoms (Figure 2). CNS involvement is often associated with hematogenous dissemination or direct extension from nearby structures [72].

In both species, disease severity is determined by factors including the parasite’s biology, host immune responses, and lesion location [69]. An early diagnosis through imaging (Figure 2), serological tests, and molecular techniques is paramount, as CNS involvement can result in neurological complications. A multidisciplinary approach is essential for an accurate diagnosis, effective management, and the potential prevention of transmission [72]. Ongoing surveillance and awareness efforts remain crucial to understanding the evolving epidemiology of echinococcosis and its impact on human and animal health in Europe [73].

#### 2.2.3. Schistosomiasis

Neuroschistosomiasis represents a significant subset of helminthic infections within the CNS. Schistosomiasis, second only to malaria in its global impact, is a paramount concern in the realm of public health and disease burden [74]. Prevalent across 74 endemic countries, schistosomiasis affects over 230 million individuals, with approximately 120 million presenting with symptomatic expressions [74,75].

The etiological agents of neuroschistosomiasis are diverse Schistosoma species, prominently encompassing *S. mansoni*, *S. haematobium*, and *S. japonicum* [76]. These parasites, intricately entwined in their life cycles involving both freshwater snails and human hosts, contribute to the persistent dissemination of the disease. The excretion of parasite eggs in urine and feces by infected individuals perpetuates the cycle of transmission [76,77].

Neuroschistosomiasis encompasses cerebral and spinal forms, characterized by the invasion of the CNS by Schistosoma parasites [74]. Clinical manifestations encompass a spectrum of neurological anomalies, including encephalopathy, myelopathy, seizures, and focal deficits [76]. The immune retort incited with the presence of schistosome eggs within the CNS culminates in granuloma formation, inflammatory processes, and consequential tissue damage [78].

Diagnostic methodologies entail the identification of schistosome eggs in urine or stool specimens, complemented with serological assays to detect specific antibodies. A cerebrospinal fluid analysis often reveals elevated protein levels and the presence of eosinophils, providing additional insights [79]. Advanced imaging techniques, notably MRI, serve as pivotal tools in visualizing granulomas and structural aberrations within the cerebral and spinal domains [78,79].

Therapeutic approaches predominantly center on praziquantel, a schistosomicidal agent effective against adult worms, supplemented with adjunctive administration of steroids to mitigate inflammatory responses [76]. 

The epidemiology of neuroschistosomiasis in Europe reveals a diverse range of cases. In France, a 28-year-old woman experienced a stroke and cerebral vasculitis 6 months after returning from Burkina Faso, occurring alongside a diagnosed *S. mansoni* disseminated infection [80]. Similarly, cases in Paris showcased encephalitis and focal neurologic deficits during acute schistosomiasis. Cerebral imaging unveiled arterial junctional territory cerebral vasculitis, potentially mediated by eosinophil-induced toxicity [81]. In the United Kingdom, a retrospective review identified four neuroschistosomiasis cases, all exhibiting symptoms of transverse myelitis. These cases were linked to freshwater exposure in Uganda, Malawi, and Nigeria [79]. Meanwhile, a patient from São Tomé and Príncipe, Portugal, displayed a successful clinical response to praziquantel therapy, marking the first reported neuroschistosomiasis case associated with that region [82]. In Spain, a male presenting myelopathy and multifocal neuritis tested positive for S. haematobium serology. This positivity supported the diagnosis, which was based on exposure history, clinical presentation, and radiological findings [83]. These instances collectively emphasize the diverse epidemiological facets of neuroschistosomiasis across Europe.

#### 2.2.4. Strongyloidiasis

Strongyloidiasis is a parasitic infection caused by the roundworm *Strongyloides stercoralis*. This infection primarily affects the intestines but can also lead to systemic effects and potential neurological complications [84].

Infection occurs when individuals come into contact with soil contaminated with Strongyloides larvae. The larvae penetrate the skin and migrate to the lungs, where they are coughed up and swallowed, eventually reaching the intestines. In some cases, the larvae can complete their life cycle within the body, leading to chronic infection. In Europe, the seroprevalence of strongyloidiasis has been reported at 9.06%, while the rate of a direct diagnosis by culture was notably lower, at 0.7% [85].

CNS strongyloidiasis represents a recognized yet uncommon form of disseminated infection. Its initial description dates back to 1973 when it was identified postmortem [86]. Nevertheless, the occurrence of CNS involvement in strongyloidiasis is exceedingly rare, particularly in non-endemic countries. A mere eight antemortem cases have been documented in such regions, reflecting the exceptional nature of this presentation [87]. Notably, the majority of these cases involve individuals with compromised immune systems, often linked to factors like corticosteroid therapy, malignancy, or chemotherapy. The prognosis for disseminated strongyloidiasis with CNS engagement remains bleak, marked with elevated mortality rates [87]. Alarmingly, the diagnosis of CNS-associated strongyloidiasis (Figure 2) is frequently postmortem, underlining the diagnostic challenges and the advanced disease state often encountered [88]. 

Diagnosing strongyloidiasis involves identifying larvae in stool samples. Serologic testing, while broadly available and sensitive, lacks specificity and can be influenced by infections with certain parasites; reduced sensitivity is observed in cases of HTLV-1 infection and hematologic malignancies [84]. The treatment approach for *S. stercoralis* infection is a matter of debate and evidence scarcity. Recommendations vary from a two-dose ivermectin regimen to single or multiple doses, particularly in uncomplicated cases, while hyper-infection requires prolonged ivermectin use with a possible antibiotic combination [87]. In CNS-involved cases, evidence remains limited, with treatments including albendazole, ivermectin, and their combination showing mixed outcomes [84].

In a case series and review conducted in France, neurological symptoms were reported in 25.6% of all cases reviewed, with a higher prevalence of 72.6% observed in the reported case series, compared to 21.2% reported in the broader literature [88]. *S. stercoralis* meningitis was documented in an immunocompromised patient of Belgian origin, illustrating the potential for infection even in temperate regions like Europe, possibly related to occupational exposure [89]. A case of Strongyloides infection involving a Brazilian man living in Portugal, who had a history of previous bacterial meningitis treated with various medications, underscores the potential complexity of CNS infections caused by *S. stercoralis* in Europe [90].

#### 2.2.5. Taeniasis (Neurocysticercosis)

Neurocysticercosis (NCC) is a parasitic infection caused by *Taenia solium* larvae in the CNS. The pathogenesis involves interactions between the parasite, host immune response, and cysticerci location. In the life cycle of *T. solium*, humans can serve as definitive hosts, harboring the adult parasite in their small intestine, while pigs, the natural intermediate host, become infected when they ingest *T. solium* eggs from improperly disposed human feces, resulting in the development of cysticerci in pig tissues. When humans consume undercooked pork containing cysticerci, they can become definitive hosts. Although it is not the typical or primary role of humans in the life cycle of *T. solium*, occasionally, humans may become intermediate hosts if they ingest eggs [91]. After ingestion, larvae invade the bloodstream, reaching the CNS where they develop into cysticerci. These bladder-like structures lodge in the brain, triggering an inflammatory response with immune cell recruitment [92]. Lymphocytes, macrophages, and eosinophils release pro-inflammatory cytokines, such as TNF-α and IL-1β, leading to the formation of an inflammatory granuloma around the cysticerci. The cysticerci’s location within the brain affects disease progression, with critical areas like the brainstem or ventricles causing more severe symptoms and complications due to disruption of neurological function [93]. 

NCC presents with diverse clinical manifestations based on lesion location and stage, primarily categorized as parenchymal and extraparenchymal NCC [94]. Parenchymal NCC often leads to seizures and chronic headaches, representing a significant cause of acquired epilepsy [5]. The evolution of cysts from viable to degenerating stages involves perilesional inflammation and can exacerbate neurological symptoms [92]. Extraparenchymal NCC, located in ventricles or subarachnoid spaces, can cause hydrocephalus and mass effects. Subarachnoid NCC is characterized by a hypercellular cyst membrane, high antigen levels, and inflammatory responses, leading to hydrocephalus and intracranial hypertension [94].

Neurocysticercosis exhibits varying clinical features influenced by factors such as cysticerci characteristics and the individual’s immune response [95]. The most common symptom is seizures, occurring in about 70% of cases and ranging in type and intensity. Headaches are frequent and result from increased pressure or inflammation. Neurological deficits such as weakness, sensory changes, visual disturbances, aphasia, or cranial nerve palsies can manifest based on the location of cysticerci [92]. Cognitive impairment, memory difficulties, personality changes, depression, or psychosis are possible. Obstruction of cerebrospinal fluid flow with cysticerci can lead to hydrocephalus, characterized by headaches, vomiting, papilledema, and altered mental status. The presentation varies among individuals, and the symptoms can overlap with other neurological conditions, making a diagnosis challenging [96]. Recognizing the diverse clinical features is crucial for the timely management of neurocysticercosis.

Even though cysticercosis is a non-notifiable disease in most European countries, human cysticercosis cases were reported in 17 western and 15 eastern European countries [97,98]. Most cases of human cysticercosis diagnosed in western Europe are associated with immigration or travel to endemic countries, particularly Latin America and the Caribbean, with a recent increase in cases from Africa and eastern Europe; however, autochthonously acquired cases in the region are rare [97]. The highest number of diagnosed human cysticercosis cases in eastern Europe was reported in Romania and Serbia, indicating potential differences in reporting and epidemiology among countries, and factors such as an underreporting, underdiagnosis, and lack of additional information hinder the understanding of parasite transmission in the region [98]. According to a study conducted in Spain, there was an initial increase in hospitalizations for neurocysticercosis from 1998 to 2008, followed by a decrease, which coincided with a decline in external migration. The most commonly associated diagnoses were epilepsy and convulsions (49.5%), hydrocephalus (11.8%), and encephalitis/myelitis/meningitis (11.6%) [99].

Recent literature highlights the diagnostic challenge posed with a limited number of laboratories in Europe that have the required tools to identify cysticercosis. These laboratories are primarily found in regions where the disease was previously endemic or where there are significant connections to endemic areas [100].

#### 2.2.6. Toxocariasis

Toxocariasis is a parasitic infection caused by the larvae of the roundworms *Toxocara canis* (from dogs) and *Toxocara cati* (from cats). While the primary effects of toxocariasis are on the visceral organs, the infection can also lead to systemic and potential neurological complications [101].

Toxocara larvae are found in the intestines of infected dogs and cats. Eggs from the worms are shed in the animals’ feces and can contaminate the environment. Humans can become infected by ingesting soil or objects contaminated with infective eggs. Once ingested, the larvae can migrate to various organs, including the liver, lungs, eyes, and CNS [102].

In some cases, toxocariasis can lead to neurological symptoms due to the migration of larvae to the CNS (Figure 2).

Neurotoxocariasis: Larvae migrating to the brain can cause neurotoxocariasis, resulting in symptoms such as headache, seizures, altered mental status, and focal neurological deficits [103].

Ocular Larva Migrans: Larvae migrating to the eyes can lead to ocular larva migrans, causing visual disturbances and inflammation of the eyes [101,102].

Diagnosing toxocariasis involves clinical evaluation, serological tests to detect antibodies (Figure 1), and imaging studies such as MRI [102]. Treatment may involve anthelminthic drugs such as albendazole or mebendazole. Management of ocular and neurological complications may require specialized care.

Epidemiological data on toxocariasis in Europe are limited, but case reports and seroprevalence studies provide insights into its occurrence. In Europe, the overall seroprevalence of toxocariasis in the general population is estimated at 6.2%, with a notable increasing trend observed over decades, reaching the highest recorded value of 12.4% in the 2010s [104]. Case reports from France have highlighted the potential for ocular and neurological complications [105]. The seroprevalence rates have been reported as 2.4% in Denmark, and 7% in Sweden [102]. These findings suggest a varying burden of toxocariasis across different European regions.

#### 2.2.7. Trichinellosis (Neurotrichinellosis)

*Trichinella* spp. is the causative agent of trichinellosis, a zoonotic infection primarily associated with the consumption of undercooked meat from infected animals. The infection is mainly contracted through the ingestion of raw or undercooked pork, game meat, or horse meat, in which Trichinella larvae encyst within the muscle tissues [106]. The consumption of these larvae initiates the infection in humans. Although Trichinella infection primarily presents with muscle-related symptoms, it has also been associated with cases of CNS involvement, referred to as neurotrichinellosis, which account for approximately 10–15% of reported trichinellosis cases [107,108]. This CNS involvement is often overshadowed by the more prevalent muscular symptoms [109].

Neurotrichinellosis presents a complex spectrum of symptoms including headache, myelitis, and cranial nerve palsies, and causes meningitis or encephalitis [108]. These neurological symptoms can accompany or follow the more common muscular symptoms of trichinellosis, such as muscle pain, fever, and edema [106]. Pathogenetic mechanisms include cerebral artery obstruction by larvae, cysts, granulomas, toxic vasculitis with secondary thrombosis, bleeding, granulomatous cerebrum inflammation, or allergic reactions [107,108,110]. Initial CNS manifestations include diffuse encephalopathy symptoms like disorientation and somnolence, progressing to focal neurological deficits such as hemiparesis [111]. Rarely, sinus venous thrombosis can occur [112].

The diagnosis of CNS involvement due to *Trichinella* spp. infection relies on a combination of factors. Clinical presentation, including neurological symptoms accompanied with severe muscle pain, should raise suspicion, particularly in regions where the infection is endemic [107,109]. Hypereosinophilia often accompanies the infection [110]. Serological tests, such as an enzyme-linked immunosorbent assay (ELISA), and Western blot (WB), are employed to detect specific antibodies against Trichinella antigens [106]. Additionally, larval identification through imaging studies can contribute to the diagnosis. Imaging commonly reveals multifocal small lesions in the cortex and white matter, which may indicate multifocal ischemic rather than inflammatory brain infiltrations [113]. In severe cases presenting with neurological symptoms, a cerebrospinal fluid analysis may reveal pleocytosis and elevated protein levels, aiding in the diagnostic process [108].

In France, nine patients displayed neurological signs such as encephalopathy and focal deficits alongside small hypodensities in the cortex and white matter on brain CT scans, with eight of them also experiencing cardiovascular events [110]. Another French case exhibited marked hypereosinophilia and subacute cortical infarcts with Gd-DTPA enhancement on MRI [113]. Romania reported a case with eosinophilia and multiple bilateral brain lesions, particularly in border zones [114]. In Germany, a patient manifested sudden-onset blindness, paralysis of the legs, and progressive arm weakness [115]. Turkish documentation identified multifocal brain lesions via MRI and diffusion-weighted MRI [116]. Lastly, in Belgrade, Serbia, a 51-year-old female with trichinellosis developed neurological symptoms, including confusion and limb weakness, with imaging revealing multiple hypodense changes in the brain corresponding to parasitic-infection-induced vasculitis [117].

**Table 2:** Epidemiology, mode of transmission, diagnosis, and treatment of helminthic infections of the CNS.

Parasites and Diseases	Countries with Reported Cases (Europe)	Mode of Transmission	Diagnosis	Treatment
*Angiostrongylus cantonensis* Angiostrongyliasis	France, Germany, the Netherlands, Switzerland, Belgium, Croatia, Italy, Spain, United Kingdom [59,62]	Ingestion of raw or undercooked molluscs, crabs, or freshwater shrimp	Radiology	Symptomatic
*Echinococcus granulosus* Cystic echinococcosis *Echinococcus multilocularis* Alveolar echinococcosis	Greece, Italy, Turkey, Romania, Bulgaria [67] Belgium, Netherlands, Italy, Austria, Hungary, and Slovenia [68]	Ingestion of eggs	Radiology, serology	Surgery, PAIR, albendazole
*Schistosoma* spp.	France, United Kingdom, Spain, Portugal [79,80,81,82,83]	Skin penetration	Demonstration of eggs in stool or urine, serology	Praziquantel
*Strongyloides stercoralis* Strongyloidiasis	France, Belgium, Portugal [88,89,90]	Skin penetration	Demonstration larvae, serology	Ivermectin
*Taenia solium* Cycticercosis	Prevalent in both eastern and western Europe [97,98,99]	Ingestion of eggs	Radiology, serology	Surgery, albendazole, and praziquantel
*Toxocara* spp. Visceral larva migrans Ocular larva migrans Neurotoxocariasis	Spain, France, Denmark, Sweden [102,104,105]	Ingestion of eggs	Serology	Albendazole
*Trichinella* spp. Trichinellosis	France, Romania, Germany, Turkey, Serbia [110,114,115,116,117]	Ingestion of raw or undercooked pork	Demonstration larvae, serology	Symptomatic, albendazole

#### 2.2.8. Other Helminthiases of CNS

While CNS involvement by the following parasites has not been reported in Europe so far, it is important to note that they also rarely cause CNS infections even in their endemic regions and on a global scale. This highlights the relatively infrequent occurrence of CNS manifestations associated with these parasites, underscoring the need for heightened vigilance and consideration of alternative etiologies in cases presenting with neurological symptoms.

##### Filariidae (*Wuchereria bancrofti*, *Brugia malayi*, *Brugia timori*, *Onchocerca volvulus*)

Filariidae infections, caused by various species including *Wuchereria bancrofti*, *Brugia malayi*, *Brugia timori*, and *Onchocerca volvulus*, are transmitted through the bites of infected mosquitoes (*W. bancrofti*, *B. malayi*, *B. timori*) or blackflies (*O. volvulus*). The mosquitoes or blackflies transfer the infective larvae (microfilariae) to humans during blood meals. These larvae then develop into adult worms, primarily residing in the lymphatic system (*W. bancrofti*, *B. malayi*, *B. timori*) or subcutaneous tissues (*O. volvulus*) [118]. The global burden of lymphatic filariasis decreased significantly from an estimated 199 million infected individuals in 2000 to approximately 51 million in 2018, with varying regional prevalence in the Americas and South East Asia [119]. Onchocerciasis, affecting a total of 37 million people, predominantly afflicts Africa, with 99% of the cases occurring on the continent [120].

While these filariidae infections primarily affect the lymphatic system (*W. bancrofti*, *B. malayi*, *B. timori*) or the skin and eyes (*O. volvulus*), CNS involvement is not a characteristic feature of these infections [121]. The pathogenesis of *Onchocerca volvulus* infection in the CNS is associated with various neurological manifestations, such as epilepsy and nodding syndrome [122]. Adult worms residing in subcutaneous nodules and the release of microfilariae can trigger inflammatory responses in the CNS. The presence of *O. volvulus* antigens and immune reactions are believed to contribute to the development of epileptic seizures [123]. Nodding syndrome, a severe neurological disorder affecting children, is also found in areas with onchocerciasis. The relationship between onchocerciasis and nodding syndrome is intricate, involving potential neuroinflammation and interactions between *O. volvulus* antigens and the immune system, which could be implicated in the syndrome’s pathogenesis [124].

The diagnosis of Filariidae infections involves identifying microfilariae in blood samples. Treatment often includes antiparasitic drugs such as diethylcarbamazine (DEC) or ivermectin, along with managing symptoms [121,125]. 

##### *Paragonimus* spp.

*Paragonimus* spp. are lung flukes transmitted to humans through the consumption of undercooked or raw freshwater crustaceans containing infective metacercariae. Once ingested, the parasites migrate to various organs, including the lungs, where they cause pulmonary symptoms [126]. Paragonimiasis has a global distribution in the Americas, Africa, and Asia, and primarily affects tropical regions, with an estimated 23 million people infected worldwide, with Asia bearing the greatest disease burden [127].

*Paragonimus* spp. can occasionally migrate to the CNS, causing cerebral paragonimiasis. However, such cases are typically more common in regions where paragonimiasis is endemic [128].

Diagnosing cerebral paragonimiasis involves clinical evaluation, imaging studies, and serological tests. Treatment often includes antiparasitic drugs such as praziquantel, along with corticosteroids to manage inflammation and alleviate symptoms [129]. 

##### Soil-Transmitted Helminths (STHs)

Soil-transmitted helminths (STHs), including *Ascaris lumbricoides*, *Trichuris trichiura*, *Necator americanus*, and *Ancylostoma duodenale*, are transmitted through ingestion of contaminated food or water (*A. lumbricoides*, *T. trichiura*) or skin penetration (*N. americanus*, *A. duodenale*) [130]. STH infections, which affect nearly one quarter of the global population and result in a loss of 5.2 million DALYs, are predominantly concentrated in tropical and subtropical regions of Sub-Saharan Africa, the Americas, and Asia, where favorable environmental conditions, socioeconomic risk factors, and limited access to safe water sources facilitate transmission [131].

STHs primarily affect the intestines and other organs, with CNS involvement not being a typical feature of these infections [75]. Diagnosing STH infections involves identifying eggs or larvae in stool samples. Treatment often includes anthelminthic drugs such as albendazole or mebendazole [75]. 

## 3. Discussion

Parasitic infections impacting the CNS are rare but impose significant morbidity and mortality [2]. The complexity of these infections and their severe consequences highlight the need for comprehensive strategies. The absence of precise diagnostic tools is a major hurdle, as these infections often present with nonspecific symptoms, causing delays in a diagnosis and treatment [3,9,132]. Their insidious nature results in missed chances for intervention, compounded with limited research and diagnostic progress [133].

Migration, climate change, and an aging population with increasing immunosuppression have reshaped the landscape of CNS parasitic infections [8,134]. Population movement introduces infections to new areas, sparking localized outbreaks, and altered climate patterns expand vector ranges, facilitating infection spread [134]. Immunocompromised individuals are more susceptible, as these infections exploit weakened immunity [17,84,93].

The zoonotic nature of these diseases necessitates a “one health” approach, understanding human–animal–environment interactions [4,10,123]. Grasping parasite life cycles and reservoir hosts is key to curbing transmission. Collaborative medical–veterinary efforts provide comprehensive insights and holistic strategies [95,135,136].

Active surveillance is crucial for identifying unusual clusters promptly, enabling swift responses [4,68]. Equally important is the education of healthcare professionals and the public. Clinicians must be able to recognize the array of clinical presentations and risk factors, while public awareness empowers individuals to take preventive measures and seek timely care [8,9]. Vital policy adjustments are needed to tackle evolving challenges, including the development of customized diagnostic tools, healthcare personnel training, and integration into existing control programs. Adapting policies to this changing landscape enhances overall healthcare responses [3].

In the context of CNS parasitic infections, critical research priorities include accurately determining the prevalence and impact of these diseases, developing user-friendly diagnostic tools, improving methods for assessing neurological, cognitive, and psychiatric consequences, advancing vaccines and preventive strategies, deepening our understanding of the disease mechanisms, refining treatments and prevention approaches for nervous system complications, conducting operational research to effectively implement established interventions, and enhancing rehabilitation methods [76]. These research areas are essential for addressing the complex challenges posed with CNS parasitic infections within the field of neuroinfectious diseases.

## 4. Conclusions

In conclusion, while CNS parasitic infections are rare, their impact on morbidity and mortality is considerable. The complex interplay of factors such as diagnostic limitations, lack of awareness, changing demographics, and zoonotic potential underscores the urgency of adopting a multidimensional approach. By fostering collaboration between medical, veterinary, and environmental sectors, enhancing surveillance and awareness, and revising policies, we can collectively address the challenges posed with these infections and minimize their devastating effects on public health.

## Data Availability

The data sets generated or analyzed during the current review are available from the corresponding author upon reasonable request. The reviewed articles, studies, and references cited in this review are publicly available through their respective journals, databases, and sources. Any additional information required to support the findings of this review can be obtained by contacting the corresponding author.
